# Low Light CMOS Contact Imager with an Integrated Poly-Acrylic Emission Filter for Fluorescence Detection

**DOI:** 10.3390/s100505014

**Published:** 2010-05-19

**Authors:** Yonathan Dattner, Orly Yadid-Pecht

**Affiliations:** Electrical and Computer Engineering, Schulich School of Engineering, University of Calgary, 2500 University Dr. NW, Calgary, AB, T2N-1N4, Canada; E-Mail: ydattner@ucalgary.ca

**Keywords:** poly-acrylic acid, CMOS contact imager, polar protic solvents, quantum efficiency, quantum yield

## Abstract

This study presents the fabrication of a low cost poly-acrylic acid (PAA) based emission filter integrated with a low light CMOS contact imager for fluorescence detection. The process involves the use of PAA as an adhesive for the emission filter. The poly-acrylic solution was chosen due its optical transparent properties, adhesive properties, miscibility with polar protic solvents and most importantly its bio-compatibility with a biological environment. The emission filter, also known as an absorption filter, involves dissolving an absorbing specimen in a polar protic solvent and mixing it with the PAA to uniformly bond the absorbing specimen and harden the filter. The PAA is optically transparent in solid form and therefore does not contribute to the absorbance of light in the visible spectrum. Many combinations of absorbing specimen and polar protic solvents can be derived, yielding different filter characteristics in different parts of the spectrum. We report a specific combination as a first example of implementation of our technology. The filter reported has excitation in the green spectrum and emission in the red spectrum, utilizing the increased quantum efficiency of the photo sensitive sensor array. The thickness of the filter (20 μm) was chosen by calculating the desired SNR using Beer-Lambert’s law for liquids, Quantum Yield of the fluorophore and the Quantum Efficiency of the sensor array. The filters promising characteristics make it suitable for low light fluorescence detection. The filter was integrated with a fully functional low noise, low light CMOS contact imager and experimental results using fluorescence polystyrene micro-spheres are presented.

## 1. Introduction

Fluorescence spectroscopy will be a key component of future micro-total-analysis-systems (μTASs) [1,2], which will integrate the capabilities of entire laboratories onto compact devices consisting of microchips and other micro-fabricated elements (Lab-on-a-chip) [3–7]. A key component in fluorescence analysis is the optical filter that separates the excitation light from the fluorescence emission. Ideally, the optical filter component should be monolithically integrated to the lab-on-a-chip device, as well as simple to fabricate at low cost [8,9].

One simple approach to building a miniaturized imaging system capable of micro scale resolution is to directly couple the sensor array with the sample of interest, referred to as *Contact Imaging* [10]. Contact image sensors, compared with conventional imagers, do not require optical elements, such as lenses between the sample and the sensor array, providing better collection efficiency without optical loss [11]. For objects in close proximity with the sensor surface, the contact imager subtends nearly 2π of the total solid angle, so the collection efficiency can be as high as 50% for samples that emit light [12]. Salama *et al*. [13] estimated that the optical efficiency of a contact imaging system is improved by 35 dB in comparison with camera-based imaging system [14]. This makes it possible to use a low power LED as an illumination source for dark objects because the improvement in collection efficiency allows the detection of a weak signal. The distance between the object of interest and the sensor array is mainly determined by the thickness of the optical filter and therefore thin filters are desired.

Fluorescence imaging is widely used in areas such as cell analysis, diagnosis bioengineering and pharmaceutical and genomic research [15–19]. Therefore the fundamental requirement of the filter is that it be bio-compatible with the object of interest. This requires minimizing the impact on cell physiology while protecting the sensor array from damage by exposure to the biological environment. Many groups have reported results for optical absorption filters using either bandgap semiconductor material or organic material [20–22]. We propose an absorption filter using poly-acrylic acid due its optical transparent properties, adhesive properties, miscibility with polar protic solvents and most importantly its bio-compatibility with a biological environment. Many groups have reported filters in the ultraviolet and blue spectrum [3,23,24]; we propose a possible filter in the red spectrum, utilizing the increased quantum efficiency of the photo sensitive sensor array.

We begin in section 2 with a brief overview of fluorescence spectroscopy to lay the groundwork for evaluating the various filter approaches. Section 3 will discuss other state of the art filter techniques and technologies. Section 4 will propose our method for design and fabrication of the optical filter. Section 5 introduces the CMOS image sensor, describes the electrical and optical setup and summarizes the figures of merit for the prototype image sensor. Section 6 will discuss the integration of the filter with the CMOS imager and show results of the complete system. Lastly, Section 7 will conclude the work.

## 2. Fluorescence Spectroscopy

Fluorescence spectroscopy, or spectrofluorometry, is a type of electromagnetic spectroscopy which analyzes fluorescence from a sample, which can be intrinsic to the specimen under study, introduced into it, or chemically bound to it. Molecules have various states referred to as energy levels. Generally, the species being examined will have a ground state (a low energy state), and an excited state of higher energy. Fluorescence, involves using a beam of light that excites a nanostructure such as an atom or a molecule to an excited state. As the nanostructure relaxes to its ground state it emits light of a lower energy, typically, but not necessarily, visible light [25]. Fluorescence spectroscopy is primarily concerned with the vibrational states.

**Figure 1.**
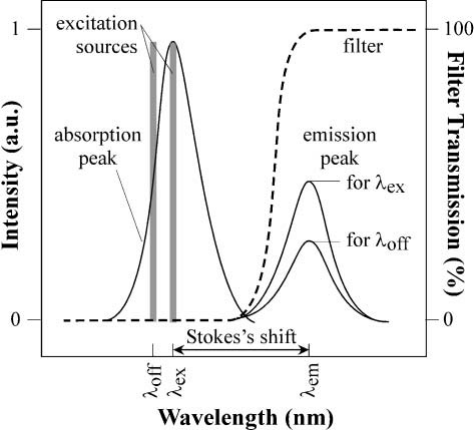
Typical peaks in the excitation (left) and emission (right) spectra, in arbitrary units. The wavelength filter (dashed line) must reject the excitation light and transmit the emitted fluorescent light. Excitation with off-peak (λ_off_) lowers the emission intensity.

The absorption spectrum illustrated for a generic fluorophore in Figure 1 has a peak at λ_ex_, and the emission spectrum has a peak at λ_em_. The distance between λ_ex_ and λ_em_ is called the *Stokes Shift*. Stokes shift can be as small 10 nm or as large as 150 nm, depending on the fluorophore [26].

If the fluorophore is excited at an off-peak wavelength *λ*_off_, the resulting fluorescence spectrum will be unchanged but will have lower amplitude then if it is excited at *λ*_ex_. The number of photons emitted is typically much smaller than the number absorbed, reflecting the existence of non-radiative pathway for the decay of the fluorophore from its excited state. The ratio of the emitted to absorbed photons is the *quantum yield* of the fluorophore. The emission light is in the order of 10^−4^ to 10^−6^ [27] of the excitation light and therefore it’s important to have high attenuation at λ_ex_ and low attenuation at λ_em_.

Fluorescence can be detected visually, for example using a fluorescence microscope, or it can be converted to an electrical signal and detected in such devices as CMOS imagers. There have been many advances in CMOS imaging in the last decade but the basic operating principle has not changed. CMOS imagers comprise of an excitation source, a wavelength filter and a detector. There are many types of excitation sources that can be used. We present our results using a Newport monochromator, which is suitable for use in laboratory conditions. The wavelength filter is of importance because it discriminates between excitation light and emission photons by significantly reducing the excitation light intensity reaching the detector while allowing through as much of the weak fluorescence signal as possible. The detector in our case is the CMOS contact imager that was designed in the Integrated Sensors, Intelligent Systems (ISIS) Lab at the University of Calgary.

Four parameters that characterize optical filters are rejection levels, transmission levels, absorption edge width or roll-off and absorbance. The rejection level is the wavelength at which wavelengths are blocked in the stop band and transmission level is the wavelength at which wavelengths are transmitted in the pass band. The absorption edge, or roll-off, is the sharpness of the transition between the stop band and the pass band. Ideally, the absorption edge should be vertical and located to the right of λ_ex_ and to the left of the entire emission spectrum. The absorbance (A = −log(*T)* = log(*I_0_/I_i_*)) is defined as minus the base 10 logarithm of the transmittance (*T* = *I_0_/I_i_*), which is the ratio of the output light intensity to the incident light intensity. Ideally, the filter should transmit 0% of the excitation light and 100% of the fluorescence emitted light. The absorbance includes losses due to absorption, reflection and scattering. Intensity is defined as power per unit area.

## 3. State of the Art Filters

The first types of filters used at the micro-scale were interference filters or dichroic filters. An interference filter consists of multiple thin layers of dielectric material having different refractive indices and there also may be metallic layers. Interference filters are wavelength-selective by virtue of the interference effects that take place between the incident and reflected waves at the thin-film boundaries. The advantages with interference filters are; compatibility with integrated circuitry which can be readily integrated into larger micro scale systems, arbitrary spectral profiles can be obtained using different layer arrangements and they can be fabricated using standard, low-temperature processes. A disadvantage of interference filters is that the spectral response depend on the angle of incidence and the polarization of the incoming light, which is a major drawback in contact imaging due to the close proximity of the object of interest with the sensor array (<100 μm). A variation of a few nanometers in the thickness of the layers can cause large errors in the cutoff wavelength which can reach ±50 nm. Another disadvantage is that it’s difficult to fabricate multiple filters of this type for different colors on one surface [26] because this would require a special process where each pixel would be covered with a different thickness of dielectric material.

*Absorption filters* are an alternative to interference filters; they are single layer filters that have high absorption at the excitation wavelength and low absorption at the emission wavelength. They are governed by Beer Lambert Law for liquids; *I* = *I*_0_*10^−ε*lc*^, where *I* is the intensity of the light after the filter, *I*_0_ is the intensity of the incident light, ɛ is the molar absorptivity of the absorber, *l* is the thickness of the filter and *c* the concentration of the absorbing species in the material.

For polymeric absorption filters there have been many demonstrated devices. Dandin *et al.* [28] demonstrated a UV-absorbing chromophore and were able to achieve −45 dB rejection of excitation wavelengths and −1.5 dB transmission of emission wavelengths on only 1.5 μm thick film. Beiderman *et al.* [29] reported a PDMS and Sudan II blue filter with −26 dB rejection of the excitation light at 340 nm and −3 dB transmission of the emission light at 450 nm for a 98 μm thick filter. Hofmann *et al.* [23] also reported a dye doped PDMS filter which resulted in 0.01% transmittance below 500 nm and >80% above 570 nm with 1 mm thick filter. Richard *et al.* [30] reported an integrated hybrid filter that incorporates both an interference and absorption filter in such a way that the advantage of each technology is used to offset the disadvantages of the other. The interference component minimizes the thickness required of the absorbing component and sharpens its roll-off characteristics while the absorbing component renders the performance of the overall filter, independent of the incidence angle. The total rejection of the hybrid filter is −43 dB at 530 nm and ∼2 dB at 650 nm with a total thickness of 2.8 μm and a roll-off of ∼100 nm.

There are many demonstrated devices that are excited in the UV-spectrum with emission in the blue spectrum, but not many are reported with excitation in the green spectrum and emission in the red spectrum. A state of the art filter should have rejection around −60 dB and transmittance close to 0 dB, with a roll-off of 20 nm or less and not thicker than a few micro-meters. A possible filter that we are reporting is excited in the green spectrum with emission in the red spectrum, taking advantage of the increased Quantum efficiency of photo detectors. We report rejection of −66 dB and transmittance of −1.6 dB with a roll-off of 20 nm using a 20 μm thick filter. The thickness can easily be improved by changing the absorbing specimen (Atrazon Orange G dye in this case) to one with a higher molar absorptivity. Due to the miscibility of poly-acrylic acid with all the polar protic solvents; water, Formic Acid, Methanol, Ethanol, Propanol, Isopropanol, Butanol and Acetic Acid; the dye can be changed and a thinner filter can be fabricated.

## 4. Emission Filters Design and fabrication

The absorption filter is based on three parts. A polar protic solvent (in this example ethyl-alcohol), an absorbing specimen (in this example Astrozan Orange G dye) and an adhesive to conform the solution into a solid filter. For an adhesive we report the poly-acrylic acid (PAA). We chose the PAA solution due its optically transparent properties when cured, it conforms to a strong non-elastic solid, its miscibility with polar protic solvents and most importantly its bio-compatibility with a biological environment [31]. It’s important that the PAA be optically transparent so we can base our analysis of the filters spectra solely on the absorbing dye particles.

The PAA is miscible with the polar protic solvents (*i.e.,* a solvent that has a hydrogen atom bound to an oxygen atom); therefore we can use solvents such as water, Formic Acid, Methanol, Ethanol, Propanol, Isopropanol, Butanol and Acetic Acid to dissolve the absorbing specimen, Astrazon Orange G dye for this specific example. We chose ethyl-alcohol, due to its high solubility with the dye; 50 mg/mL. After the solvent is mixed with the dye, 1ml of solution is added to 1ml of PAA and the filter is left to cure until the ethyl-alcohol fully evaporates.

According to Beer Lambert’s Law for liquids absorbance becomes a linear equation:

A=ε*l*c

**Figure 2.**
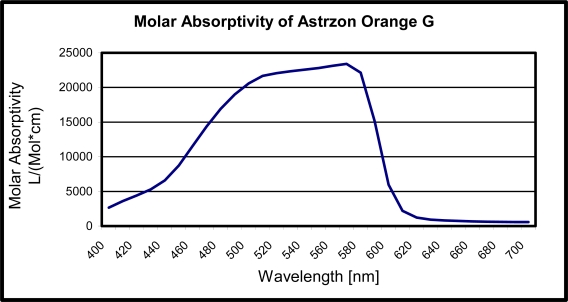
Molar Absorptivity of Astrazon Orange G dye as a function of wavelength.

Where A is the absorbance, *l* is the thickness of the filter and *c* is the concentration of absorbing species in the material. Therefore once a dye is chosen, the two parameters left in the filter design are the concentration and the thickness. The molar absorptivity of the Astrazon Orange G dye as a function of wavelength is displayed in Figure 2.

The Astrazon Orange G dye dissolves best in the Ethyl-Alcohol solvent; we can achieve a concentration of 50 mg/ml. Any larger concentrations produce dye aggregation which will cause non-uniformity to the filter. To calculate the desired thickness of our filter we define SNR as the ratio of number of electrons produced from the fluorophore to the number of electrons produced due to excitation light in our CMOS sensor array for one pixel. To simplify calculation we assume the excitation light has a Gaussian profile (*i.e.,* the transverse electric field and intensity distributions are described by Gaussian functions), the fluorophore is a point source with isotropic emission light and we neglect any optical path losses after the filter to the sensor array. Under these assumptions we can calculate the minimum filter’s thickness to give us the desired SNR.

To determine the light power density in *W / m^2^*:

$$I\left(\frac{W}{m^{2}}\right)=\Phi *\frac{\mathrm{hc}}{\lambda }(J)=q\Phi \frac{1.24}{\lambda (um)}$$

With the photon flux is defined as:

$$\Phi =\frac{\#\text{of}\;\text{photons}}{{\text{sec}*m}^{2}}$$

From Beer Lambert’s law we can derive that transmittance *T* is:

$$T=\frac{I_{i}}{I_{0}}=10^{-\varepsilon lc}$$

The fluorescence quantum yield is defined as the ratio of the number of photons emitted to the number of photons absorbed. The quantum yield for most flourophores is between 10^−4^ to 10^−6^, to calculate our SNR we’ll take the worst case of 10^−6^.

The quantum efficiency:

$${QE}_{\lambda }=\eta =\frac{N_{E}}{N_{\nu }}$$

**Figure 3.**
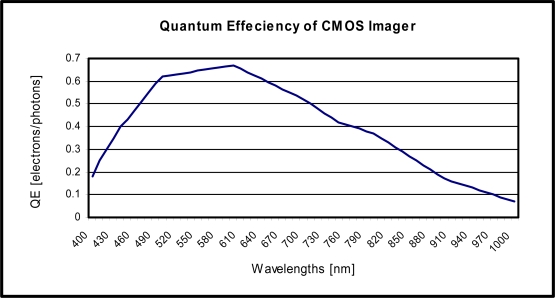
Quantum Efficiency of an n-well over p-substrate photodiode in the prototype CMOS imager.

Where *N_e_* = number of electrons produced, *N_ν_* = number of photons absorbed. The filter is integrated on a CMOS contact imager explained in the next section and therefore we will be using the measured QE to calculate the filters SNR. The measured quantum efficiency as a function of wavelength is shown in Figure 3. We can notice in Figure 3 that the QE is highest in the red spectrum. Many filters have been reported in the UV and blue spectrum for excitation and emission, respectively, but few have reported absorption filters in the red spectrum. Our specific example of the combination of Astrazon Orange G and Ethyl alcohol used with PAA produces a red filter and therefore utilizes the increased QE of the sensor array.

**Figure 4.**
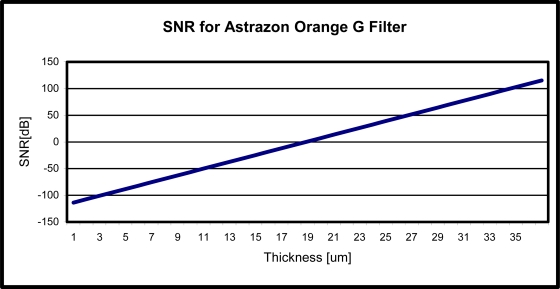
Calculated SNR as a function of the filters thickness.

Using this information, we can see in Figure 4 that a minimum filter thickness of 20 μm is needed to achieve a positive SNR. For contact imaging, the maximum distance that the object under test can be from the sensor array is 100 μm. If the object is farther than 100 μm, contrast degradation becomes a major issue [12]. Therefore, our design of a 20 μm thick filter will satisfy the required maximum distance for contact imaging.

**Figure 5.**
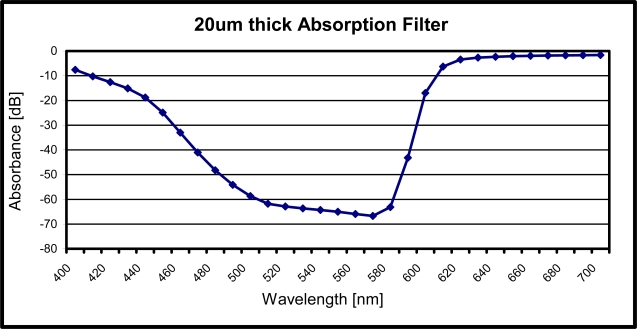
Absorbance of a 20 μm thick filter with a concentration of 50 mg/mL of Astrazon Orange G dye dissolved in Ethyl-Alcohol. The filter is hardened using the Poly-Acrylic Acid as a bio-compatible and optically transparent adhesive.

Using the calculations from above and Beer Lamberts law, the filters absorbance spectra can be derived as shown in Figure 5. The filter has max attenuation of −66 dB at 570 nm and minimum attenuation of −1.6 dB at 650 nm. With a roll-off of 20 nm the filter allows good contrast imaging and is suitable for low light fluorescence detection.

## 5. Measurements of the Image Sensor

**Figure 6.**
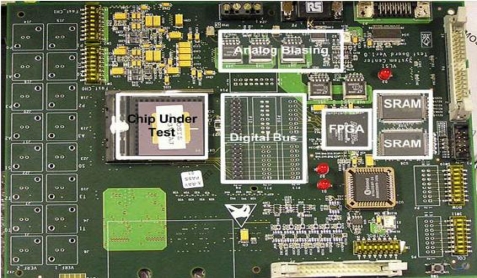
Versatile board for mixed signal applications.

A prototype 128 × 128 image sensor chip was fabricated in a six-metal, single-poly, mixed signal CMOS TSMC 0.18 μm process and operates with a 1.8 V supply. The sensor array is designed with a pixel pitch of 7 μm. The pixel utilizes an n-well over p substrate photodiode and achieves a fill factor of 30%. As described in [29], the chip design employs Active Reset (AR) and Active Column Sensor (ACS) readout techniques for low noise operation allowing low light imaging. This makes the prototype design suitable for fluorescent applications in which the signal is usually very weak. The main difference is that this version of the chip employs the best pixel from [29], in terms of dark current, and is used for the whole sensor array. Furthermore this version has a new package allowing compatibility with the versatile board. The fast versatile board for mixed signal applications shown in Figure 6 includes a cyclone II FPGA for control of digital signals and data, 12 bit ADC to convert the analog pixel voltage to a digital signals, SRAM to store the frames, analog biasing for the CMOS imager and many other functions to sufficiently test the prototype image sensor. There are many advantages of using the versatile board for testing the imager, but one of the most important benefits is that less noise is introduced to the system then the previous setup reported in [29].

The figures of merit for the prototype image sensor are summarized below in Table 1.

## 6. Filter Integration and Results

Using a room temperature vulcanizing (RTV) silicon sealant by Vishay^®^ (M-Coat C), all the exposed parts of the CMOS imager can be covered apart from the sensor array. This material was selected mainly due to its higher viscosity and rapid curing properties, in addition to its chemical resistance to many solvents, see Figure 6.

**Figure 7.**
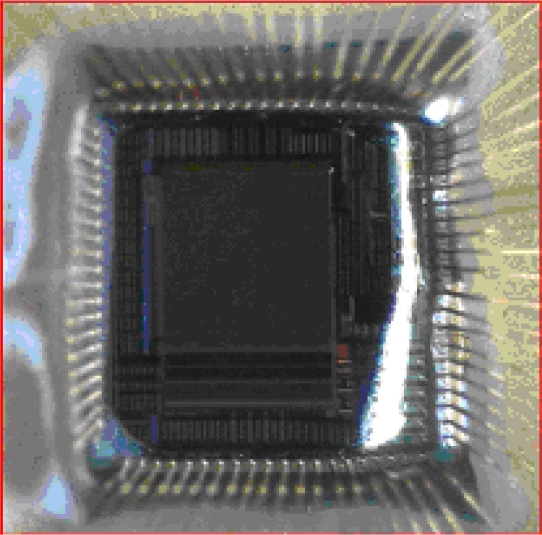
CMOS Contact Imager coated with RTV silicon Sealant, apart from the sensor array.

Figure 7 depicts the cross section of the system, in which the CMOS Imager is wire bonded with a PGA108M package so that it can be tested with the versatile board. The RTV sealant is applied with a fine brush to the exposed parts covering the wire bonds and overlapping part of the CMOS Imager chip, but it does not cover any part of the sensor array. The PAA filter is poured on top of the sensor array and is encapsulated by the RTV sealant. The filter is spun in a 1000 clean room and left to cure until all the solvent evaporates. Once the filter hardens the bio-material under test can be placed on the surface.

**Figure 8.**
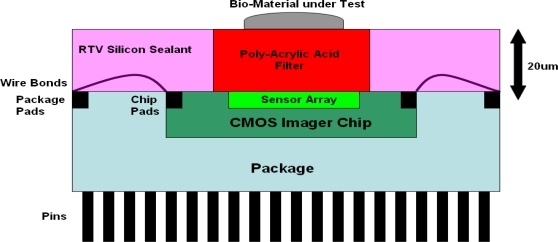
Cross section of the CMOS imager with the integrated PAA absorption filter.

Fluorescent polystyrene micro-spheres approximately 40 μm in diameter and non-fluorescent micro-spheres approximately the same size where placed on the image sensor with the integrated filter using a 10-μL micropipette. The fluorescent micro-spheres are excited with green light (∼542 nm) and emit red light (∼610 nm.). For these wavelengths the filter absorbs −63 dB of the excitation light and −6 dB of the emission light. Figure 8a shows an image of the fluorescence and non-fluorescence micro-spheres seen under a conventional microscope (top view of sensor array, using incoherent light source without employing florescence imaging). The fluorescence micro-spheres can be identified as they are brighter under the incoherent light source while the non-fluorescence micro-spheres are darker. Figure 8b depicts the same scene, as captured by the sensor array after picture enlargement and contrast adjustments. The fluorescence micro-spheres emit red light and therefore color was added to Figure 8b for illustration purposes.

## 7. Conclusion

This paper provides a description of a fully functional low-light 128 × 128 prototype CMOS contact imager with a newly developed process for an integrated emission filter using poly-acrylic acid for fluorescence detection. Various fluorescence applications can be employed using the present process, while we showed a specific combination that produced a red filter, the absorbing specimen and solvent can be changed and filters in different parts of the spectrum can easily be produced using the same process. The filter described in section 4 produced promising results with a 20 nm roll-off, high transmission in the pass-band and high absorbance in the stop-band, and therefore is suitable for low light fluorescence detection. The hardened filter is poly-acrylic acid based, and due to its bio- compatibility the process is suitable for bio-sensing applications.

## Figures and Tables

**Figure 9.**
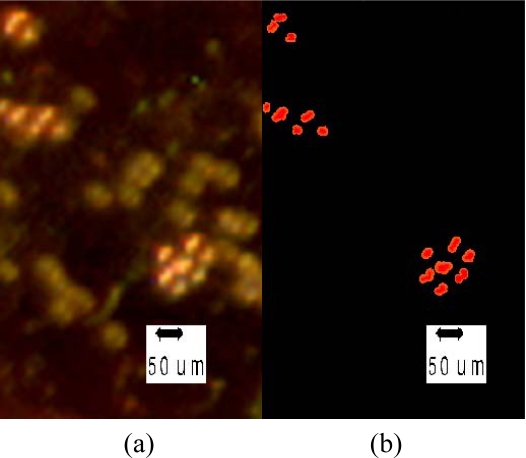
Fluorescence micro-spheres (Brighter beads in (a)) and non-fluorescence micro-spheres (Darker beads in (a)) were placed on the emission filter and excited with green light (∼500 nm–560 nm). Image of the micro-spheres (a) the chip micrograph (b) the sensor array after picture enlargement and contrast adjustments. Color was added for illustration purposes.

**Table 1.** Image Sensor Performance Figures of Merit.

**Parameter**	**Measurement**
Array Size	128 × 128
Pixel Size	7 μm · 7 μm
Supply Voltage	1.8 V
Fill Factor	30%
Conversion Gain	29 μV/e^−^
Dark Current Density (worst case)	31 nA/cm^2^
Pixel FPN (reset frame)	0.16%
Column FPN (reset frame)	0.04%
Peak QE	29%
QE (at 610 nm)	29%
Readout Non-Linearity	0.6%
Reset Noise	9.2 e^−^
Operation rate	30 fps
Partitioned Amplifier Gain	66 dB
